# Gene Expression Profiling of Human Decidual Macrophages: Evidence for Immunosuppressive Phenotype

**DOI:** 10.1371/journal.pone.0002078

**Published:** 2008-04-30

**Authors:** Charlotte Gustafsson, Jenny Mjösberg, Andreas Matussek, Robert Geffers, Leif Matthiesen, Göran Berg, Surendra Sharma, Jan Buer, Jan Ernerudh

**Affiliations:** 1 Division of Clinical Immunology, Unit for Autoimmunity and Immune Regulation (AIR), Department of Clinical and Experimental Medicine, Faculty of Health Sciences, University Hospital, Linköping, Sweden; 2 Department of Clinical Microbiology, County Hospital Ryhov, Jönköping, Sweden; 3 Mucosal Immunity, Helmholtz Centre for Infection Research (HCI), Braunschweig, Germany; 4 Division of Gynecology and Obstetrics, Faculty of Health Sciences, University Hospital, Linköping, Sweden; 5 Department of Pediatrics, Brown University and Women and Infants' Hospital of Rhode Island, Providence, Rhode Island, United States of America; 6 Department of Pathology, Brown University and Women and Infants' Hospital of Rhode Island, Providence, Rhode Island, United States of America; 7 Institute of Medical Microbiology, University Hospital Essen, Essen, Germany; New York University School of Medicine, United States of America

## Abstract

**Background:**

Although uterine macrophages are thought to play an important regulatory role at the maternal-fetal interface, their global gene expression profile is not known.

**Methodology/Principal Findings:**

Using micro-array comprising approximately 14,000 genes, the gene expression pattern of human first trimester decidual CD14+ monocytes/macrophages was characterized and compared with the expression profile of the corresponding cells in blood. Some of the key findings were confirmed by real time PCR or by secreted protein. A unique gene expression pattern intrinsic of first trimester decidual CD14+ cells was demonstrated. A large number of regulated genes were functionally related to immunomodulation and tissue remodelling, corroborating polarization patterns of differentiated macrophages mainly of the alternatively activated M2 phenotype. These include known M2 markers such as CCL-18, CD209, insulin-like growth factor (IGF)-1, mannose receptor c type (MRC)-1 and fibronectin-1. Further, the selective up-regulation of triggering receptor expressed on myeloid cells (TREM)-2, alpha-2-macroglobulin (A2M) and prostaglandin D2 synthase (PGDS) provides new insights into the regulatory function of decidual macrophages in pregnancy that may have implications in pregnancy complications.

**Conclusions/Significance:**

The molecular characterization of decidual macrophages presents a unique transcriptional profile replete with important components for fetal immunoprotection and provides several clues for further studies of these cells.

## Introduction

A distinctive immune tolerance environment is programmed at the maternal-fetal interface to avoid rejection by the maternal immune system. The most abundant maternal immune cells in the decidualized endometrium are NK cells and macrophages. Although their exact roles are currently being debated, they are thought to regulate vascular growth and placenta development. Global gene expression profiling of decidual NK cells provided a unique pattern compared to their counterparts in blood [1]. In the case of monocytes/macrophages, accumulating data indicate that local environment drives them into functionally different subsets. Classically, macrophages activated by agents like IFN-γ, TNF or bacterial LPS, show profound effector functions like production of toxic intermediates, essential in the killing of intracellular microbes. They also secrete high amounts of pro-inflammatory and Th1 cytokines such as TNF and IL-12. On the other hand, macrophages activated by Th2 cytokines such as IL-4 and IL-13 as well as anti-inflammatory cytokine IL-10 induce a suppressive mode of activation and have been termed alternatively activated macrophages ([2], reviewed in [3], [4]). In this regard, Mantovani *et al* [5] proposed a nomenclature where M1 and M2 represent blood derived macrophages activated by inflammatory and anti-inflammatory cytokines, respectively. However, it is not clear whether decidual macrophages represent mainly M1 or M2 phenotype.

Due to their role in immune regulation, macrophages in the uterus are likely to contribute to local immune tolerance in normal pregnancy. They may do so by phagocytosis of apoptotic cells harbouring potentially pro-inflammatory and harmful effects on the fetus (reviewed in [6]). Temporal expression of anti-inflammatory cytokines such as IL-4 [7]–[10], IL-13 [11]–[13] and IL-10 [7]–[10], [14], [15] is evident at the maternal-fetal interface, suggesting an environment capable of facilitating alternative activation of macrophages. It is thus likely that decidual macrophages reveal a suppressive or regulatory phenotype in the placenta [16], [17] as well as in the decidua [9], [18]–[21]. We report here, for the first time, a global gene expression pattern of decidual monocytes/macrophages. We compared them with the expression patterns of blood CD14 positive cells. A clearly distinct gene expression profile in decidual macrophages is evident, confirming a selective gene cascade regulation involved in immune modulation and tissue remodelling.

## Materials and Methods

### Subjects

Decidual tissue and blood was obtained from eleven women between 18 and 41 years of age (median age 35 years) with normal pregnancies, undergoing elective surgical abortions in week 7 to 11 (median week 9) at the department of Gynecology and Obstetrics, Linköping University Hospital, Sweden. All pregnancies were detected viable and dated by crown-rump length measurement with ultrasound. Misoprostol (Cytotec®; Searle, USA) was given to four of the eleven subjects before the procedure; the data from these subjects did not differ from the other subjects. Laboratory analyses performed in the different subjects are shown in table 1. Written informed consent was obtained from all subjects. The study was approved by the Local Ethics Committee of Linköping University.

**Table 1:** Methods performed on the different subjects

Subject	FACSAria sorting	MACS sorting	Micro-array	Real-time PCR	Protein detection	Flow cytometry*
1	X		X			X
2	X		X			X
3	X			X		X
4	X			X		X
5	X			X		X
6		X	X			X
7		X	X		X	X
8		X	X		X	X
9		X	X		X	
10		X	X		X	
11		X			X	

* Flow cytometry for control of purity

### Isolation of CD14 positive cells and extraction of total RNA

Mononuclear cells were separated from decidua and blood as described previously [9]. CD14 positive cells were obtained by two different methods. Cells from five subjects were sorted by FACSAria cell sorting, a method resulting in high cell purity but with the possible disadvantage of causing high mechanical stress to the cells. Positive immunomagnetic MACS separation, a fast method with a relatively low physical impact on the cells, was performed in the six remaining subjects.

For immunomagnetic separation, CD14 positive cells were selected with anti-CD14 antibody coated beads (Miltenyi Biotec, Bergish Gladbach, Germany) according to the manufacturer's instructions. Flow cytometry in 3 of 6 samples showed purity (% CD14^+^ cells) of 84, 88 and 92 in blood and 72, 74 and 76 in the decidua, respectively. For FACSAria (BD Bioscience) cell sorting, cells were labelled with anti-CD4FITC, CD14APC (Miltenyi Biotech), CD45PE-Cy5 and CD3 PE-Cy7 antibodies (BD Bioscience, Stockholm, Sweden). CD14 positive cells were defined as CD45^+^CD3^−^CD14^+^ cells and sorted accordingly. Purity of FACSAria sorted CD14 positive cells in the two subjects used for micro-array was 98.0 to 99.8% in blood and 96.2–97.2% in decidua, respectively. In the three subjects used for real-time PCR, purity was 93–98% in blood and 86–96% in the decidua, respectively. Following sorting, cells were lysed in RNeasy RLT lysing buffer and total RNA was extracted according to the manufacturer's instructions (Qiagen, West Sussex, UK).

### Affymetrix GeneChip Assay

Samples were amplified for GeneChip analysis according to the recommended protocols by the manufacturer (Affymetrix, Santa Clara, CA, USA). In all cases, 10 µg of each biotinylated cRNA preparation was fragmented and placed in a hybridization cocktail containing four biotinylated hybridization controls (BioB, BioC, BioD, and Cre), as recommended by the manufacturer. Samples were hybridized to an identical lot of Affymetrix HG U133 2.0 GeneChips for 16 hours. After hybridization the GeneChips were washed, stained with SA-PE, and read using an Affymetrix GeneChip fluidic station and scanner.

### Data Analysis

Analysis of micro-array data was performed using the Affymetrix GCOS 1.2 software. For normalization all array experiments were scaled to a target intensity of 150, otherwise using the default values of GCOS 1.2. Further downstream analysis was performed using Array Assist 4.0 (Stratagene, La Jolla, CA, USA). Data was normalized by the PLIER algorithm (Affymetrix, Santa Clara, CA, USA) using default parameters. Genes whose signal maximum intensity did not exceed 100 across all samples were excluded from further analysis. Student's T-Test was applied to identify differences between decidual and blood monocytes/macrophages. Genes whose p values were below or equal to 0.05 and mean fold changes more/less than +2/−2 fold in cells from two of the high purity FACSAria separated subjects were considered differentially regulated in decidual samples compared with blood cells. These genes were used as a gene expression signature for the experiment, resulting in 408 regulated genes. Among these, genes fulfilling the same criteria in cells from the five MACS separated subjects were selected and resulted in the final 120 regulated genes. Alongside with these high-stringent requirements, data analysis with lower stringency was performed. With the requirement of two-fold increase/decrease and statistical difference in all seven subjects considered as a common group with no respect to the separation method used, a much higher number of genes were revealed regulated (n∼1700). We chose to further analyse the genes obtained by the high-stringency requirements. These genes were grouped according to plausible functions in the context of macrophages in early pregnancy, resulting in the groups immune modulation, tissue remodelling, cell cycle related and cell metabolism/transport.

### Real-time PCR

To confirm regulated genes in the micro-array, real-time PCR was performed on flow-cytometrically sorted CD14 positive cells obtained from three additional subjects. RNA was reversely transcribed with 500 U SuperScript II reverse transcriptase (Invitrogen, Carlsbad, California) according to the manufactureŕs protocol. Table 2 provides all genes with corresponding primers (synthesized by TIB Molbiol, Berlin, Germany) used for real-time PCR. Relative quantification was performed using the 7500 Fast Real-Time PCR System (Applied Biosystems, Foster City, California) under the following conditions: 10 min at 95 °C followed by 40 cycles at 95°C for 15seconds and 60 seconds at 60°C. Amplification of specific PCR products was detected using the SYBR Green PCR Master Mix (Applied Biosystems) in duplicates. The housekeeping ribosomal protein S9 (RPS9) gene was used for normalization. The number of genes analyzed was restricted by the low amount of lysate available.

**Table 2:** Primers used for real-time RT-PCR

Product	5′ Sequence 3′	Funktion	Product length	T_m_
IDO	ATT TGT CTG GCT GGA AAG GCA ACC	sense	121 bp	59,9 °C
	AAG CAC TGA AAG ACG CTG CTT TGG	antisense		59,9 °C
TREM-2	ACA ACT TGT GGC TGC TGT CCT T	sense	122 bp	57,3 °C
	TTC CGC AGC GTA ATG GTG AGA GT	antisense		59,5 °C
CD 209	AAA TCA GGA AGG CAC GTG GCA ATG	sense	86 bp	59,9 °C
	TGT TGG GCT CTC CTC TGT TCC AAT	antisense		59,9 °C
ICAM-3	CAA TCT CAG CAA CGT GAC TGG CAA	sense	90 bp	59,9 °C
	ACG GTG ATG TTA GAG GAG CCT GTT	antisense		59,9 °C
NRP-1	AAG ACC TTC TGC CAC TGG GAA CAT	sense	103 bp	59,9 °C
	AGT TGC CAT CTC CTG TGT GAT CCT	antisense		59,9 °C
RPS-9	GGC GCA GAC GGT GGA AGC	sense	83 bp	59,6 °C
	GGT CTC CGC GGG GTC ACA T	antisense		60,0 °C

T_m_: melting temperature

### Analysis of ex vivo protein secretion from CD14 positive cells

Blood and decidual immunomagnetically separated CD14 positive cells (50 000 cells) from five subjects were incubated over night in 200 µL of tissue culture medium (TCM) consisting of Iscoves modification of Dulbecco's media (Gibco BRL, Paisley, Scotland) supplemented with (given as final concentrations in the medium): L-glutamine (Flow Laboratories, Irvine, Scotland) 292 mg/L; sodium bicarbonate 3.024 g/L; penicillin 50 IE/mL and streptomycin 50 µg/mL (Flow Laboratories) and 100 x non essential amino acids 10 mL/L (Flow Laboratories). The cell-free supernatants were stored at −70°C. The protein level of CCL18 was determined by an in-house double-antibody sandwich ELISA using a monoclonal anti-human CCL18 (0.5 µg/mL;clone 64507; R&D Systems, Abingdon, UK) and a polyclonal biotinylated anti-human CCL18 Antibody (200 ng/ml; R&D Systems). Streptavidin-poly-horse radish peroxidase and 3,3′,5,5′-tetramethylbenzidine liquid substrate (Sigma-Aldrich) were used for detection (Sandberg *et al*, unpublished results). The levels of CCL2 and matrix metalloproteinase (MMP)-9 in cell culture supernatants were analyzed according to the manufacturer's instructions. The kits used were base kits LUH000 and LMP000 together with analyte kits LUH279 and LMP911 (all purchased fromR&D Systems, Abingdon, UK). Protein concentrations were analyzed using the Luminex 100 instrument (Luminex Corporation, Austin, Texas, USA) and the STarStation software (v 2.3, Applied cytometry Systems, Sheffield, UK). Genes were selected for protein analyses based on significance and possibility of detection due to availability of assay.

### Online supplemental material

A complete table of the 120 differentially regulated genes is provided as supplementary material (Table S1). The entire microarray dataset is available at GEO database under Acc. GSE10612 (http://www.ncbi.nlm.nih.gov/projects/geo/).

## Results

### Micro-array data

**Figure 1:**
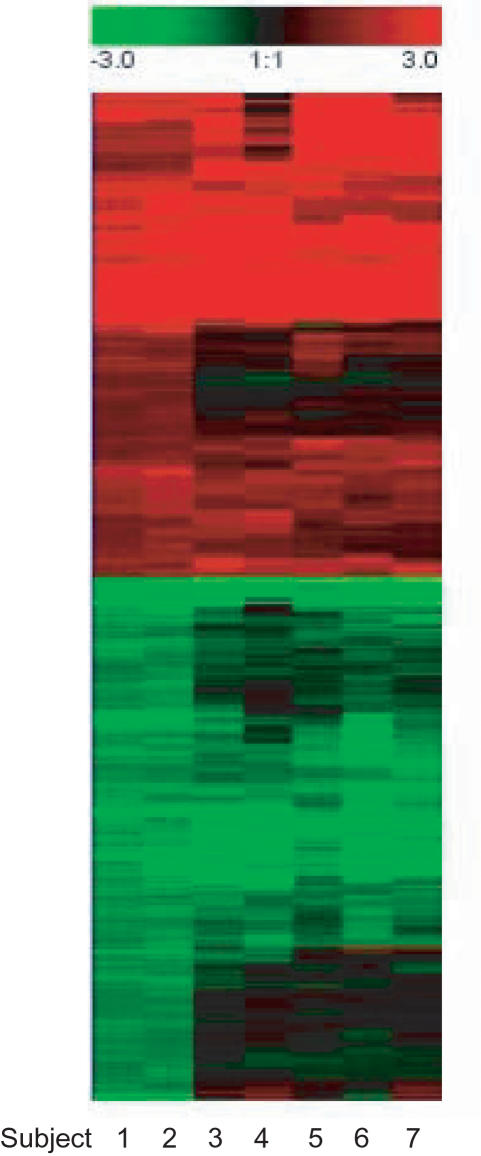
Cluster figure of the gene signature of 408 genes regulated in decidual compared to blood macrophages, showing all seven subjects (columns). Requirements for regulation was 2-fold up-/down-regulation and a p-value <0.05 in the two subjects with highly pure cellpopulations (subjects 1 and 2).

Gene expression data revealed a total of 120 genes differently regulated (80 up-regulated and 40 down-regulated) in decidual CD14 positive cells compared to their blood counterparts, when using the most stringent statistical criteria. A complete list of differentially regulated genes is provided as supplementary material and a selection is summarized in Table 3. A major group of genes encode for proteins with immune regulatory functions. Further, genes associated with tissue remodelling, cell proliferation and cell metabolism were commonly regulated. Figure 1 shows a cluster picture of the gene signature of 408 regulated genes.

**Table 3:** Selection of genes differentially expressed in decidual compared to blood CD14^+^ cells in early pregnancy.

Gene name					Gene bank accession number	Fold change^a^
**Immune modulating**						
		Suppressive/Anti-inflammatory functions				
			*Soluble form*			
				Alpha-2-macroglobulin; A2M	NM_000014	92
				V-set and immunoglobulin domain containing 4;VSIG4	NM_007268	23
				Prostaglandin D2 synthase, hematopoietic; PGDS	NM_014485	13
				Chemokine (C-C motif) ligand 18;CCL18, AMAC1	Y13710	3.8
			*Membrane bound form*			
				Mannose receptor, C type 1;MRC1	NM_002438	36
				CD209 antigen; DC-SIGN	AF290886	16
				Triggering receptor expressed on myeloid cells 2;TREM2	NM_018965	9.0
				Tumor necrosis factor receptor superfamily, member 21; TNFRSF21	NM_016629	7.3
				CD9 antigen (p24); CD9	NM_001769	5.3
				Dipeptidase 2;DPEP2	NM_022355	−2.8
				C-type lectin domain family 4, member A;CLEC4A (LLIR)	AF200738	−3.0
			*Intracellular protein*			
				Disabled homolog 2, mitogen-responsive phosphoprotein (Drosophila); DAB2	AF188298	30
				V-maf musculoaponeurotic fibrosarcoma oncogene homolog (avian); MAF	NM_005360	12
				Interleukin-1 receptor-associated kinase 3;IRAK3	NM_007199	−2.7
		Activating/Pro-inflammatory functions				
			*Soluble form*			
				Secreted phosphoprotein; SPP1	M83248	101
				Complement component 3;C3	NM_000064	48
				Chemokine (C motif) ligand 2; XCL2	U23772	7.6
				Chemokine (C-C motif) ligand 2; CCL2, MCP1	S69738	5.8
				Chemokine (C motif) ligand 1; XCL1 (lymphotactin)	NM_003175	5.7
				Chemokine (C-C motif) ligand 8;CCL8, MCP2	AI984980	5.0
				Proteolipid protein 2 (colonic epithelium-enriched);PLP2	NM_002668	−4.0
				S100 calcium binding protein A8 (calgranulin A);S100A8	NM_002964	−5.8
				Properdin P factor, complement;PFC	NM_002621	−6.6
			*Membrane bound form*			
				SLAM family member 8;SLAMF8	NM_020125	4.7
				Bone marrow stromal cell antigen 1;BST1 (CD157)	NM_004334	−3.1
				Asialoglycoprotein receptor 2;ASGR2, CLEC4H2	NM_001181	−4.1
				Intercellular adhesion molecule 3;ICAM3	NM_002162	−4.8
				EGF-like-domain, multiple 5;EGFL5	W68084	−5.9
				Selectin L (lymphocyte adhesion molecule 1);SELL	NM_000655	−7.0
				Vanin 2;VNN2	NM_004665	−7.5
**Tissue remodelling**						
				Fibronectin 1;FN1	AF130095	68
				Complement component 1, q subcomponent, beta polypeptide;C1QB	NM_000491	58
				Collagen, type III, alpha 1;COL3A1	AU144167	27
				Heat shock 27kDa protein 1; HSPB1	NM_001540	21
				Complement component 1, q subcomponent, alpha polypeptide;C1QA	NM_015991	16
				Endothelial PAS domain protein 1;EPAS1	AF052094	15
				Growth arrest-specific 6;GAS6	L13720	11
				Collagen, type I, alpha 2;COL1A2	AA788711	10
				Collagen, type VI, alpha 3;COL6A3	NM_004369	9.9
				Collagen, type IV, alpha 2;COL4A2	X05610	9.3
				Serpin peptidase inhibitor, clade F, member 1;SERPINF1	NM_002615	8.2
				Nerve growth factor receptor (TNFRSF16) associated protein 1;NGFRAP1	NM_014380	7.9
				Matrix metalloproteinase 9;MMP9, plasminogen	NM_004994	6.5
				Protein S (alpha);PROS1	NM_000313	5.3
				Insulin-like growth factor 1 (somatomedin C);IGF1	AI972496	5.1
				Syndecan 2 (heparan sulfate proteoglycan 1);SDC2	AL577322	4.4
				Integrin, beta 5;ITGB5	BE138575	3.7
				S100 calcium binding protein A4 (calcium protein, calvasculin, metastasin, murine placental homolog);S100A4	NM_002961	−2.8
				Chondroitin sulfate proteoglycan 2 (versican);CSPG2	BF590263	−3.2
				Peptidyl arginine deiminase, type IV;PADI4	NM_012387	−5.9
**Cell cycle related**						
				Glycoprotein (transmembrane) nmb;GPNMB	NM_002510	54
				Ribonucleotide reductase M2 polypeptide;RRM2	BC001886	38
				Thymidylate synthetase;TYMS	NM_001071	7.1
				Ankyrin repeat domain 25;ANKRD25	NM_015493	5.5
				Deafness, autosomal dominant 5;DFNA5	NM_004403	5.0
				CDC20 cell division cycle 20 homolog (S. cerevisiae);CDC20	NM_001255	4.6
				KIAA0101	NM_014736	3.8
				Enolase superfamily member 1;ENOSF1	NM_017512	3.7
				Pituitary tumor-transforming 1;PTTG1	NM_004219	3.4
				MCM2 minichromosome maintenance deficient 2, mitotin (S. cerevisiae);MCM2	NM_004526	3.2
				Epithelial membrane protein 1;EMP1	NM_001423	3.1
				MCM6 minichromosome maintenance deficient 6 (MIS5 homolog, S. pombe) (S. cerevisiae);MCM6	NM_005915	3.0
				Hypothetical protein MAC30;MAC30	BF038366	2.8
				Sprouty homolog 2 (Drosophila);SPRY2	NM_005842	2.8
				Ribonuclease, RNase A family, 1 (pancreatic);RNASE1	NM_002933	2.8
				Stathmin 1/oncoprotein 18;STMN1	NM_005563	2.7
				Cyclin D3;CCND3	NM_001760	−2.9
				Ras association (RalGDS/AF-6) domain family 2;RASSF2	NM_014737	−2.9
				Chromosome 11 open reading frame 21;C11orf21	NM_014144	−3.0
				Cytidine deaminase;CDA	NM_001785	−4.4
**Cell metabolism/transport**						
				Selenoprotein P, plasma, 1;SEPP1	NM_005410	73
				Apolipoprotein E;APOE	NM_000041	43
				Phospholipid transfer protein;PLTP	NM_006227	30
				Solute carrier organic anion transporter family, member 2B1;SLCO2B1	NM_007256	21
				Folate receptor 2 (fetal);FOLR2	NM_000803	12
				Phosphatidic acid phosphatase type 2B;PPAP2B	AA628586	11
				Ectonucleotide pyrophosphatase/phosphodiesterase 2;ENPP2	D45421	10
				Phosphatidic acid phosphatase type 2A;PPAP2A	AF014403	7.5
				Dehydrogenase/reductase (SDR family) member 3;DHRS3	NM_004753	6.5
				Solute carrier family 2 (facilitated glucose transporter), member 1;SLC2A1	NM_006516	4.2
				ATP-binding cassette, sub-family A (ABC1), member 1;ABCA1	NM_005502	3.6
				Chromosome 9 open reading frame 95/Nicotinamide riboside kinase;C9orf95, NRK1	NM_017881	2.8
				Hexokinase 3 (white cell);HK3	NM_002115	−2.7
				Purinergic receptor P2X, ligand-gated ion channel, 1;P2RX1	U45448	−2.7
				RAB11 family interacting protein 1 (class I);RAB11FIP1	NM_025151	−3.2
				MOCO sulphurase C-terminal domain containing 1;MOSC1	NM_022746	−3.5
				Solute carrier organic anion transporter family, member 3A1;SLCO3A1	NM_013272	−3.5
				RAB27A, member RAS oncogene family;RAB27A	U38654	−4.0
				Selenoprotein X, 1;SEPX1	NM_016332	−4.0
				Transketolase (Wernicke-Korsakoff syndrome);TKT	L12711	−4.0

a Fold change is the factor of regulation of mRNA from CD14^+^ cells in decidua versus CD14^+^ cells in blood. Positive values denote up-regulation and negative values mean down-regulation. Genes included in this table were up- or down-regulated by a factor of at least 2 in all seven subjects.

### Real-time PCR for confirmation of mRNA expression

**Figure 2:**
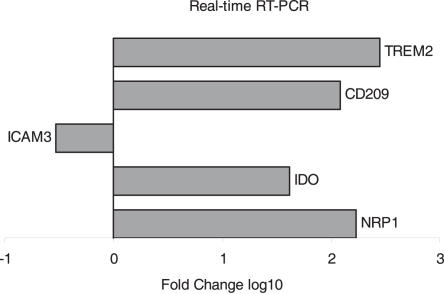
Mean results from three independent real-time PCR analyses for TREM2, CD209, ICAM3, IDO and NRP-1 showing fold change of mRNA expression in decidual compared to blood CD14 positive cells.

Real-time PCR was used to further confirm the expression of three genes differentially expressed in the array, triggering receptor expressed on myeloid cells (TREM-2), CD209 and ICAM-3. Further, two genes, indoleamine 2,3-dioxygenase (IDO) and neuropilin (NRP-1), with two-fold regulation in all seven subjects but just below statistical significance were analyzed. PCR results showed that these five genes were differently regulated in decidual CD14 positive cells compared to cells from blood (Figure 2).

### Luminex and ELISA for confirmation of protein secretion

**Figure 3:**
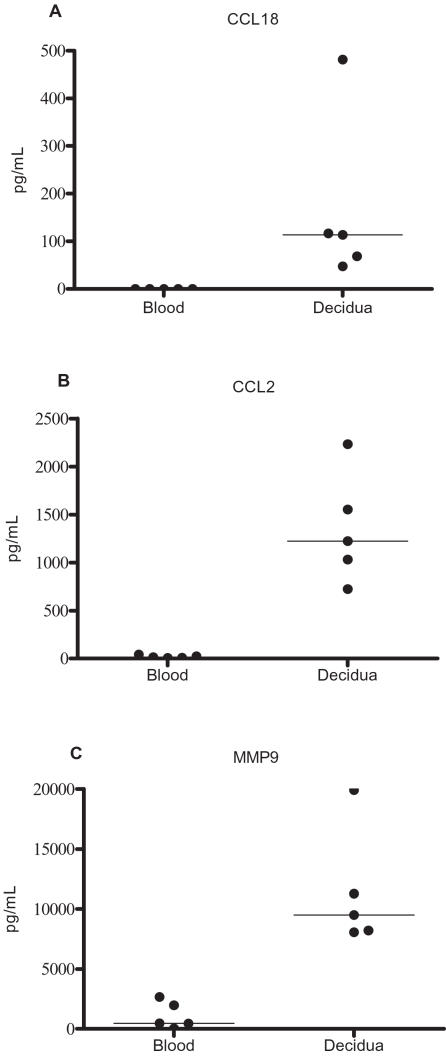
Protein concentrations of a) CCL18 as measured with ELISA, b) CCL2 and c) MMP9 as measured by Luminex multiple bead technology in 24 hour culture supernatant of CD14 positive monocytes/macrophages isolated from 5 subjects. Error bars show median values and p<0.01 for differences between decidual and blood CD14 positive cells for all proteins, using Wilcoxon signed ranked test.

Altered gene expression observed in the array analysis was also studied at the protein level by assaying 24-hour cell culture supernatant of CD14 positive macrophages. Decidual CD14 positive cells secreted significantly higher levels of CCL-18, CCL2 and MMP-9 compared to blood monocytes/macrophages, in agreement with the array data (Figure 3).

## Discussion

The maternal-fetal interface presents a unique immune site with the decidua populated with numerous and specialized NK cells and macrophages. Here we report a distinct and well characterized micro-array analysis of human decidual macrophage gene expression, comprising immunomodulatory and tissue remodelling genes, as well as genes related to cell proliferation and metabolism. While only few up-regulated genes were signature of classically activated M1 macrophage phenotype [22]–[24], several of the regulated genes corresponded to markers of alternatively activated macrophages, including CCL18 [25], CD209 [26], mannose receptor C type (MRC)-1 [3], fibronectin-1 [3] and insulin-like growth factor (IGF)-1 [25]. All of these were recently shown to be up-regulated in blood derived M2 macrophages in a gene expression comparison with M1 polarized macrophages [22]. In addition, a number of the differentially regulated genes have previously been assigned to decidual macrophages and are confirmed by our data. We also report evidence of other markers connected to alternative macrophage activation, e.g. TREM-2, alpha 2 macroglobulin (A2M) and prostaglandin D_2_ synthase (PGDS).

Among the up-regulated genes a major group could be classified as immune modulatory with immuno-suppressive or anti-inflammatory functions. In agreement with this, a group of activating or pro-inflammatory genes were down-regulated in decidual macrophages compared to their blood counterparts. Several of the up-regulated genes are surface receptors, among these the often co-expressed lectins MRC-1 (CD206, macrophage mannose receptor; MMR) and CD209 as well as the tetraspanin CD9. All have previously been detected in uterine cells in human pregnancy [1], [27]–[31]. MRC1 and CD209 (dendritic cell specific intercellular adhesion molecule-grabbing nonintegrin; DC-SIGN) are generally associated with alternative activation profile of macrophages. NRP-1, involved in establishment of the immunological synapse, is a receptor connected to regulatory T cells [32]. NRP-1 is up-regulated in decidual macrophages and was recently shown to be up-regulated in M2 cells [22], indicating a potential role in macrophage polarization.

TREM-2 expression, to our knowledge, has not been previously reported in uterine tissue or in relation to pregnancy. In this regard, we propose a novel mechanism of potential importance of TREM-2 in maternal-fetal tolerance. In mouse macrophages, TREM-2 is induced by IL-4 and is down-regulated by IFN-γ or LPS [33]. Information on TREM-2 in humans is very limited, although it was found to be down-regulated in LPS-stimulated human monocyte-derived dendritic cells (DCs) [34]. Murine macrophages express a ligand for TREM-2 on their surface thus enabling auto-regulation [35]. High expression of TREM-2 in mouse microglial cells correlate with their ability to phagocytose apoptotic neurons, hence TREM-2 might be positively regulating phagocytosis but negatively regulating inflammatory responses [36]. This proposed dual function of TREM-2 positive cells would fit well in the context of the pregnant uterus, where the need for both immune regulation and apoptotic clearance is high.

Chemokines play a central role in polarization of macrophages. CCL2 and CCL18 were found to be up-regulated, the latter being involved in recruitment and possibly tolerance indcuction of naïve T cells [37]. Interestingly, CCL18 was found to be expressed in human decidual and amnion tissues during pregnancy whereas expression was reduced after onset of labour [38]. On the other hand, CCL2 (monocyte chemoattractant protein-1; MCP-1) is classically linked with inflammatory responses (reviewed in [39]). However, CCL2 was shown to drive Th2 polarization in mice and stimulation with IL-4/IL-13 in human lung epithelial cells increased their secretion of CCL2 [40]. Daly *et al* hypothesized a dual effect of CCL2 depending on environmental demands [39]. We therefore suggest a role for CCL2 in pregnancy in recruiting monocytes/macrophages to the decidua, where other local factors will be decisive for polarization. PGDS is another up-regulated soluble immunemodulating molecuble that has been associated with decidual Th2 cell recruitment and antigen presentation [41]. IDO, a tryptophan depleting enzyme, was ascribed a role in murine pregnancy [42]. Although the role of IDO at the maternal-fetal interface has been questioned [43], its expression in decidual macrophages has previously been noted [19], thus a finding confirmed in this study.

Considering the plasticity and growth of the feto-placental unit, functions related to angiogenesis and depletion of apoptotic cells are important for a successful pregnancy. Accordingly, a large group of genes up-regulated in decidual CD14 positive cells was associated with tissue remodelling. Fibronectin increases apoptotic clearance of cells coated e.g. with C1Qs, both molecules being up-regulated here. We also found up-regulation of collagen genes as well as of other molecules promoting clearance or angiogenesis, e.g. growth arrest-specific (GAS)-6 and protein S alpha (PROS1). Although MMP9 (plasminogen) is considered a proinflammatory chemokine, it also cleaves denatured collagens and type IV collagens in basement membranes, thereby contributing to remodelling of extracellular matrix and migration of immune cells. A2M regulates functions of cytokines but in pregnancy it is suggested to particularly be involved in tissue remodelling during trophoblast invasion [44], [45]. IGF-I is an angiogenic factor that affects fetal nutrition and size (reviewed in [46] and its expression is associated with alternatively activated macrophages [22], [47], in agreement with the angiogenic characteristics of this cell type. We here confirm the role of uterine macrophages in tissue remodelling and angiogenesis during placental invasion [6], [48], and extend knowledge by adding a number of regulated genes involved in tissue remodelling.

A large group of regulated genes was related to cell cycle functions. Taking into consideration the balance between up- and down-regulated genes, the net effect was a clear-cut signature of proliferation, which is in line with the previously demonstrated proliferative capacity of macrophages [49], [50]. Our results also closely resemble the array data from Martinez *et al* [22] where many genes associated with cell proliferation were up-regulated in macrophage differentiation and priming of M1 as well of M2.Genes related to cell metabolism and transport were common both in the up- and down-regulated groups of genes. This is in line with Martinez *et al* where for example lipid metabolites and solute carriers were regulated in differentiation and priming of macrophages [22].

While the present study focused on analysis of the gene expression profile, previous studies have reported cell surface expression of CD14^+^ decidual macrophages to include CD209 [29], [30], HLA-DR [19], [29] and CD68 [19], whereas the relative expression of CD80, CD83 and CD86 was lowered [19], [29], indicating a potential to induce T-cell anergy. Previous studies have also shown a suppression of mixed leukocyte reaction (MLR) as well as of mitogen-induced proliferation of decidual macrophages as compared with their blood counterparts, thus linking decidual macrophages to a functional suppressive phenotype [21].

Macrophages show a high degree of plasticity and they adapt to the tissue environment [51], often developing properties that do not precisely fit into the in vitro generated M1 or M2 profiles, e.g. macrophages in lung tissue [52] or in tumors (reviewed in [53]). Although decidual macrophages mainly fall into the M2 category, it is important to note that they do exhibit a unique profile. The precise requirements for this polarization remain to be settled, but local cytokines and hormones are likely to have important roles. We also speculate for a role of hepatocyte growth factor (HGF) since the gene expression profile of monocytes cultured with HGF [54] resembled that of decidual CD14 positive cells reported here. HGF is normally expressed at high levels in the placenta whereas low levels are associated with pregnancy complications [55]–[57]. Polarized macrophages do not represent stable lineages, but rather show reversible adaptations to changes of the environment [51], [52], [58]. Thus, while the unique profile of decidual macrophages is important in fetal protection, an alteration of this gene expression could be, in part, involved in complications of pregnancy. Indeed, a number of genes regulated in this array were previously found to be associated with pregnancy complications such as preeclampsia (MR [28], CCL2 [28], IGF-1 [46], secreted phosphoprotein (SPP)-1 [59], MMP-9 [60]), preterm labour (CCL18 [38]) and intrauterine growth restriction (IGF-1 [46]).

Purity of cell populations is extremely important in gene expression profiling. Our strategy of using gene expression in the highly purified samples as a requirement for further consideration resulted in high-stringent criteria minimizing the risk of reporting contaminating genes. The distinction between macrophages and dendritic cells in the decidua is not clear-cut. Dendritic cells may express the macrophage related CD14 [30] whereas macrophages may express the DC related CD209. [29], [30]. On the other hand, CD14+ decidual cells were shown to express HLA-DR, CD68 and CD209, but were negative in CD1a, CD83 and CD86 [29] and, in addition, CD14+ cells were not able to differentiate into DCs when cultured in GM-CSF and IL-4 [19], thus indicating that CD14+ decidual cells mainly resemble macrophages. In any case, the expression profile of the major population of CD14 cells is of relevance irrespective of its relation to dendritic cells.

In conclusion, we present here the global gene expression pattern of decidual CD14 positive macrophages. The up-regulation of cell cycle genes indicates a highly proliferative nature of decidual cells. Although a number of nuclear factors were regulated, compatible with the differentiation process of decidua infiltrating macrophages, the main block of the regulated genes represented membrane receptors or secreted proteins, pointing to a profound polarization. The expression profile, with a large number of regulated genes related to immunomodulation and tissue remodelling, mainly parallels that of M2 polarized macrophages, including M2 markers such as CCL18, CD209, IGF-1, MRC-1 and fibronectin-1. Further, the up-regulation of for example TREM2, A2M and PGDS provide new insights into the regulating function of macrophages in pregnancy, and several genes might also be implicated in immune tolerance in general. Interestingly, some of the regulated genes described here for normal pregnancies, have been shown to be dysregulated in complicated pregnancies. Taken together, the mapping of decidual CD14 positive macrophages showed a unique transcriptional profile that confirms and extends previous knowledge about these cells as important components of fetal protection.

## Supporting Information

Table S1 Genes differentially expressed in decidual compared to blood CD14 positive cells in early pregnancy.
(0.12 MB DOC)
