# Supplementary material for: Gene Expression Profiling of Human Decidual Macrophages: Evidence for Immunosuppressive Phenotype
Source: PLoS One. 2008 Apr 30;3(4):e2078. doi: 10.1371/journal.pone.0002078 (PMC2323105; doi:10.1371/journal.pone.0002078)
Supplement: Table S1 — Genes differentially expressed in decidual compared to blood CD14 positive cells in early pregnancy. (0.12 MB DOC) [file pone.0002078.s001.doc]

| **Table S1.** Genes differentially expressed in decidual compared to blood CD14^+^ cells in early pregnancy. | | | | | | |
| --- | --- | --- | --- | --- | --- | --- |
| **Gene name** | | | | | **Gene bank accession number** | **Fold change*** |
|  | | | | |  |  |
| Secreted phosphoprotein; SPP1 | | | | | M83248 | 101 |
| Alpha-2-macroglobulin; A2M | | | | | NM_000014 | 92 |
| Selenoprotein P, plasma, 1;SEPP1 | | | | | NM_005410 | 73 |
| Fibronectin 1;FN1 | | | | | AF130095 | 68 |
| Complement component 1, q subcomponent, beta polypeptide;C1QB | | | | | NM_000491 | 58 |
| Glycoprotein (transmembrane) nmb;GPNMB | | | | | NM_002510 | 54 |
| Complement component 3;C3 | | | | | NM_000064 | 48 |
| Apolipoprotein E;APOE | | | | | NM_000041 | 43 |
| Ribonucleotide reductase M2 polypeptide;RRM2 | | | | | BC001886 | 38 |
| Mannose receptor, C type 1;MRC1 | | | | | NM_002438 | 36 |
| Granulysin;GNLY | | | | | M85276 | 34 |
| Disabled homolog 2, mitogen-responsive phosphoprotein (Drosophila); DAB2 | | | | | AF188298 | 30 |
| Phospholipid transfer protein;PLTP | | | | | NM_006227 | 30 |
| Collagen, type III, alpha 1;COL3A1 | | | | | AU144167 | 27 |
| V-set and immunoglobulin domain containing 4;VSIG4 | | | | | NM_007268 | 23 |
| Heat shock 27kDa protein 1; HSPB1 | | | | | NM_001540 | 21 |
| Solute carrier organic anion transporter family, member 2B1;SLCO2B1 | | | | | NM_007256 | 21 |
| Peripheral myelin protein 22;PMP22 | | | | | L03203 | 19 |
| CD209 antigen; DC-SIGN | | | | | AF290886 | 16 |
| Complement component 1, q subcomponent, alpha polypeptide;C1QA | | | | | NM_015991 | 16 |
| Endothelial PAS domain protein 1;EPAS1 | | | | | AF052094 | 15 |
| Prostaglandin D2 synthase, hematopoietic; PGDS | | | | | NM_014485 | 13 |
| V-maf musculoaponeurotic fibrosarcoma oncogene homolog (avian); MAF | | | | | NM_005360 | 12 |
| Folate receptor 2 (fetal);FOLR2 | | | | | NM_000803 | 12 |
| Growth arrest-specific 6;GAS6 | | | | | L13720 | 11 |
| Phosphatidic acid phosphatase type 2B;PPAP2B | | | | | AA628586 | 11 |
| Collagen, type I, alpha 2;COL1A2 | | | | | AA788711 | 10 |
| Ectonucleotide pyrophosphatase/phosphodiesterase 2;ENPP2 | | | | | D45421 | 10 |
| Granzyme A (granzyme 1, cytotoxic T-lymphocyte-associated serine esterase 3) ;GZMA | | | | | NM_006144 | 10 |
| Interleukin 2 receptor, beta;IL2RB | | | | | NM_000878 | 10 |
| Collagen, type VI, alpha 3;COL6A3 | | | | | NM_004369 | 9.9 |
| Collagen, type IV, alpha 2;COL4A2 | | | | | X05610 | 9.3 |
| Killer cell lectin-like receptor subfamily C, member 1/2;KLRC1/2 | | | | | NM_002260 | 9.2 |
| Triggering receptor expressed on myeloid cells 2;TREM2 | | | | | NM_018965 | 9.0 |
| T cell receptor alpha locus;TRA@/TRD@ | | | | | X06557 | 8.6 |
| Serpin peptidase inhibitor, clade F, member 1;SERPINF1 | | | | | NM_002615 | 8.2 |
| Matrix-remodelling associated 7;MXRA7 | | | | | BF968134 | 8.2 |
| Nerve growth factor receptor (TNFRSF16) associated protein 1;NGFRAP1 | | | | | NM_014380 | 7.9 |
| Chemokine (C motif) ligand 2; XCL2(lymphotactin) | | | | | U23772 | 7.6 |
| Phosphatidic acid phosphatase type 2A;PPAP2A | | | | | AF014403 | 7.5 |
| Collectin sub-family member 12;COLEC12 | | | | | NM_030781 | 7.4 |
| Tumor necrosis factor receptor superfamily, member 21; TNFRSF21 | | | | | NM_016629 | 7.3 |
| Thymidylate synthetase;TYMS | | | | | NM_001071 | 7.1 |
| Homeodomain-only protein;HOP | | | | | AB059408 | 7.0 |
| Putative insulin-like growth factor II associated protein;LOC492304 | | | | | X07868 | 6.6 |
| Matrix metalloproteinase 9;MMP9 | | | | | NM_004994 | 6.5 |
| Dehydrogenase/reductase (SDR family) member 3;DHRS3 | | | | | NM_004753 | 6.5 |
| Chemokine (C-C motif) ligand 2; CCL2 (MCP1) | | | | | S69738 | 5.8 |
| Chemokine (C motif) ligand 1; XCL1 | | | | | NM_003175 | 5.7 |
| Ankyrin repeat domain 25;ANKRD25 | | | | | NM_015493 | 5.5 |
| Perforin 1 (pore forming protein);PRF1 | | | | | AI445650 | 5.4 |
| CD9 antigen (p24); CD9 | | | | | NM_001769 | 5.3 |
| Protein S (alpha);PROS1 | | | | | NM_000313 | 5.3 |
| Insulin-like growth factor 1 (somatomedin C);IGF1 | | | | | AI972496 | 5.1 |
| Chemokine (C-C motif) ligand 8;CCL8, MCP2 | | | | | AI984980 | 5.0 |
| Deafness, autosomal dominant 5;DFNA5 | | | | | NM_004403 | 5.0 |
| SLAM family member 8;SLAMF8 | | | | | NM_020125 | 4.7 |
| Immunoglobulin superfamily, member 4;IGSF4 | | | | | NM_014333 | 4.7 |
| CDC20 cell division cycle 20 homolog (S. cerevisiae);CDC20 | | | | | NM_001255 | 4.6 |
| Lipoma HMGIC fusion partner-like 2;LHFPL2 | | | | | N66633 | 4.5 |
| Syndecan 2 (heparan sulfate proteoglycan 1);SDC2 | | | | | AL577322 | 4.4 |
| Solute carrier family 2 (facilitated glucose transporter), member 1;SLC2A1 | | | | | NM_006516 | 4.2 |
| Sema domain, immunoglobulin domain (Ig), transmembrane domain (TM) and short cytoplasmic domain, (semaphorin) 4C;SEMA4C | | | | | AI949392 | 3.9 |
| Chemokine (C-C motif) ligand 18;CCL18, AMAC1 | | | | | Y13710 | 3.8 |
| KIAA0101 | | | | | NM_014736 | 3.8 |
| Leprecan-like 1;LEPREL1 | | | | | NM_018192 | 3.8 |
| Integrin, beta 5;ITGB5 | | | | | BE138575 | 3.7 |
| Enolase superfamily member 1;ENOSF1 | | | | | NM_017512 | 3.7 |
| ATP-binding cassette, sub-family A (ABC1), member 1;ABCA1 | | | | | NM_005502 | 3.6 |
| Pituitary tumor-transforming 1;PTTG1 | | | | | NM_004219 | 3.4 |
| MCM2 minichromosome maintenance deficient 2, mitotin (S. cerevisiae);MCM2 | | | | | NM_004526 | 3.2 |
| Nucleolar and spindle associated protein 1;NUSAP1 | | | | | NM_016359 | 3.2 |
| Epithelial membrane protein 1;EMP1 | | | | | NM_001423 | 3.1 |
| MCM6 minichromosome maintenance deficient 6 (MIS5 homolog, S. pombe) (S. cerevisiae);MCM6 | | | | | NM_005915 | 3.0 |
| Hypothetical protein MAC30;MAC30 | | | | | BF038366 | 2.8 |
| Sprouty homolog 2 (Drosophila);SPRY2 | | | | | NM_005842 | 2.8 |
| Ribonuclease, RNase A family, 1 (pancreatic);RNASE1 | | | | | NM_002933 | 2.8 |
| Chromosome 9 open reading frame 95 / Nicotinamide riboside kinase;C9orf95, NRK1 | | | | | NM_017881 | 2.8 |
| Stathmin 1/oncoprotein 18;STMN1 | | | | | NM_005563 | 2.7 |
| Septin 11;SEPT11 | | | | | AL534972 | 2.7 |
| Benzodiazapine receptor (peripheral);BZRP | | | | | NM_000714 | -2.5 |
| Glia maturation factor, gamma;GMFG | | | | | NM_004877 | -2.5 |
| Interleukin-1 receptor-associated kinase 3;IRAK3 | | | | | NM_007199 | -2.7 |
| Hexokinase 3 (white cell);HK3 | | | | | NM_002115 | -2.7 |
| Purinergic receptor P2X, ligand-gated ion channel, 1;P2RX1 | | | | | U45448 | -2.7 |
| S100 calcium binding protein A4 (calcium protein, calvasculin, metastasin, murine placental homolog);S100A4 | | | | | NM_002961 | -2.8 |
| Dipeptidase 2;DPEP2 | | | | | NM_022355 | -2.8 |
| Cyclin D3;CCND3 | | | | | NM_001760 | -2.9 |
| Ras association (RalGDS/AF-6) domain family 2;RASSF2 | | | | | NM_014737 | -2.9 |
| C-type lectin domain family 4, member A;CLEC4A (LLIR) | | | | | AF200738 | -3.0 |
| Chromosome 11 open reading frame 21;C11orf21 | | | | | NM_014144 | -3.0 |
| Bone marrow stromal cell antigen 1;BST1 (CD157) | | | | | NM_004334 | -3.1 |
| Chondroitin sulfate proteoglycan 2 (versican);CSPG2 | | | | | BF590263 | -3.2 |
| RAB11 family interacting protein 1 (class I);RAB11FIP1 | | | | | NM_025151 | -3.2 |
| CD244 natural killer cell receptor 2B4;CD244 | | | | | NM_016382 | -3.3 |
| Myotubularin related protein 11;MTMR11 | | | | | NM_006697 | -3.3 |
| DKFZP434C171 protein;DKFZP434C171 | | | | | AL080169 | -3.4 |
| Hypothetical protein CG003;13CDNA73 | | | | | NM_023037 | -3.4 |
| Kelch repeat and BTB (POZ) domain containing 11;KBTBD11 | | | | | NM_014867 | -3.4 |
| KIAA0513 | | | | | NM_014732 | -3.4 |
| MOCO sulphurase C-terminal domain containing 1;MOSC1 | | | | | NM_022746 | -3.5 |
| Solute carrier organic anion transporter family, member 3A1;SLCO3A1 | | | | | NM_013272 | -3.5 |
| Ribonuclease, RNase A family, 2 (liver, eosinophil-derived neurotoxin);RNASE2 | | | | | NM_002934 | -3.6 |
| Eukaryotic translation initiation factor 2C, 2;EIF2C2 | | | | | AW971415 | -3.6 |
| Colony stimulating factor 3 receptor (granulocyte);CSF3R | | | | | NM_000760 | -3.8 |
| Proteolipid protein 2 (colonic epithelium-enriched);PLP2 | | | | | NM_002668 | -4.0 |
| RAB27A, member RAS oncogene family;RAB27A | | | | | U38654 | -4.0 |
| Selenoprotein X, 1;SEPX1 | | | | | NM_016332 | -4.0 |
| Transketolase (Wernicke-Korsakoff syndrome);TKT | | | | | L12711 | -4.0 |
| Asialoglycoprotein receptor 2;ASGR2 (CLEC4H2) | | | | | NM_001181 | -4.1 |
| Cystatin A (stefin A);CSTA | | | | | NM_005213 | -4.2 |
| Cytidine deaminase;CDA | | | | | NM_001785 | -4.4 |
| Intercellular adhesion molecule 3;ICAM3 | | | | | NM_002162 | -4.8 |
| Chromosome 6 open reading frame 32;C6orf32 | | | | | NM_015864 | -4.8 |
| S100 calcium binding protein A8 (calgranulin A);S100A8 | | | | | NM_002964 | -5.8 |
| EGF-like-domain, multiple 5;EGFL5 | | | | | W68084 | -5.9 |
| Peptidyl arginine deiminase, type IV;PADI4 | | | | | NM_012387 | -5.9 |
| Properdin P factor, complement;PFC | | | | | NM_002621 | -6.6 |
| Selectin L (lymphocyte adhesion molecule 1);SELL | | | | | NM_000655 | -7.0 |
| Vanin 2;VNN2 | | | | | NM_004665 | -7.5 |
|  |  |  |  |  |  |  |
| *Fold change is the factor of regulation of mRNA from CD14^+^ cells in decidua versus CD14^+^ cells in blood. Positive values denote up-regulation and negative values mean down-regulation. Genes included in this table were up- or down-regulated by a factor of at least 2 in all seven subjects. | | | | | | |
